# A novel Al matrix composite reinforced by nano-AlN_p_ network

**DOI:** 10.1038/srep34919

**Published:** 2016-10-10

**Authors:** X. Ma, Y. F. Zhao, W. J. Tian, Z. Qian, H. W. Chen, Y. Y. Wu, X. F. Liu

**Affiliations:** 1 Key Laboratory for Liquid–Solid Structural Evolution & Processing of Materials, Ministry of Education, Shandong University, Jinan 250061, China; 2 College of Materials Science and Engineering, Chongqing University, Chongqing 400044, China

## Abstract

In pursuit of lightweighting of automobiles and low emission of transportation, the efforts to develop high-strength, heat-resistant and fatigue-resistant Al alloys and/or composites have been ongoing. Here we report a novel Al matrix composite with ultrahigh strength reinforced by a three dimensional network of nano-AlN particles for the first time. The *in-situ* synthesized AlN particles are connected by twinning bonding chains and built up a three dimensional network strengthening Al matrix enormously like the skeleton to human body. The composite containing 16.4wt.% AlN particles shows excellent properties: the ultimate tensile strengths can be up to 518MPa at room temperature and 190MPa at 350 °C. This peculiar performance results from the novel spatial distribution of nano-scale AlN particles. Our findings in this work would help to develop a potential candidate for high-performance heat resistance light-metal based materials.

Owing to the positive combination of low density, high specific strength and elastic modulus, aluminum matrix composites with designed properties are becoming increasingly widely used in the fields such as aerospace, automotive engine, electronic packaging, precision instruments and sports equipment, etc^1^,^2^,^3^,^4^,^5^. To meet the requirement in some special fields, the ultrahigh strength of composites at high temperatures is crucial^6^,^7^. Currently, the most commonly used heat-resistant Al alloy includes A319^8^,^9^. However, the properties of A319 drop sharply with elevating temperatures for the coarsening and harmful phase transition of metastable intermetallic compounds. In order to solve the problem, some Al matrix composites have been fabricated by using dispersed ceramic particles like Si_3_N_4_^10^ or Al_2_O_3_^11^,^12^. For particles reinforced composites, the properties can be affected by many important factors including particulate types^13^, sizes^14^,^15^,^16^, volume fractions^17^,^18^ and the interfaces between matrix and secondary phases^19^. Another non-negligible aspect is correlated to the spatial distribution of strengthening phases^20^,^21^,^22^,^23^. Hard ceramic particles can strengthen the grain boundaries and help to achieve higher strength than current base alloys. As a kind of refractory ceramic, AlN has excellent comprehensive properties such as high thermal conductivity (320 W•m^−1^K^−1^), high elastic modulus (310GPa, 1090 °C), low coefficient of thermal expansion (4.4 × 10^−6^ K^−1^, 25 ~ 400 °C) and a relatively low density of 3.26 g/cm^3^, therefore it has great potential to be a superb candidate for fabricating heat resistant composites^24^,^25^,^26^,^27^. Extensive studies have been done on the mechanical properties of AlN_p_ reinforced metal-matrix composites at room temperature (RT)^28^,^29^,^30^,^31^. Among them, Li^28^ prepared the AlN particles (AlN_p_)/Al composites, which incorporated AlN_p_ in the matrix through a combined method of wet mixing, cold isostatic pressing and hot extrusion. The ultimate tensile strength (UTS) of this material reached 310MPa at RT. Reddy^29^ investigated an AlN_p_ *in-situ* reinforced aluminum composite using a gas bubbling method with nitrogen gas as the gaseous precursor while pure aluminum as matrix. Balog^30^ synthesized the AlN_p_/Al composites by a sinter-aluminum-pulver method and achieved good mechanical properties.

According to the Hansen-Shtrikman (H-S) bounds theory^31^, the mechanical properties of materials containing multi-phases material can be improved greatly via adjusting the distribution of the reinforcement. In this work, based on the upper H-S bounds principle, a novel Al-based composite has been specially designed by the *in-situ* construction of three-dimensional (3D) AlN_p_ network.

## Results

### Phases identification and microstructural characterization of the AlN_p_/Al composites

**Figure 1:**
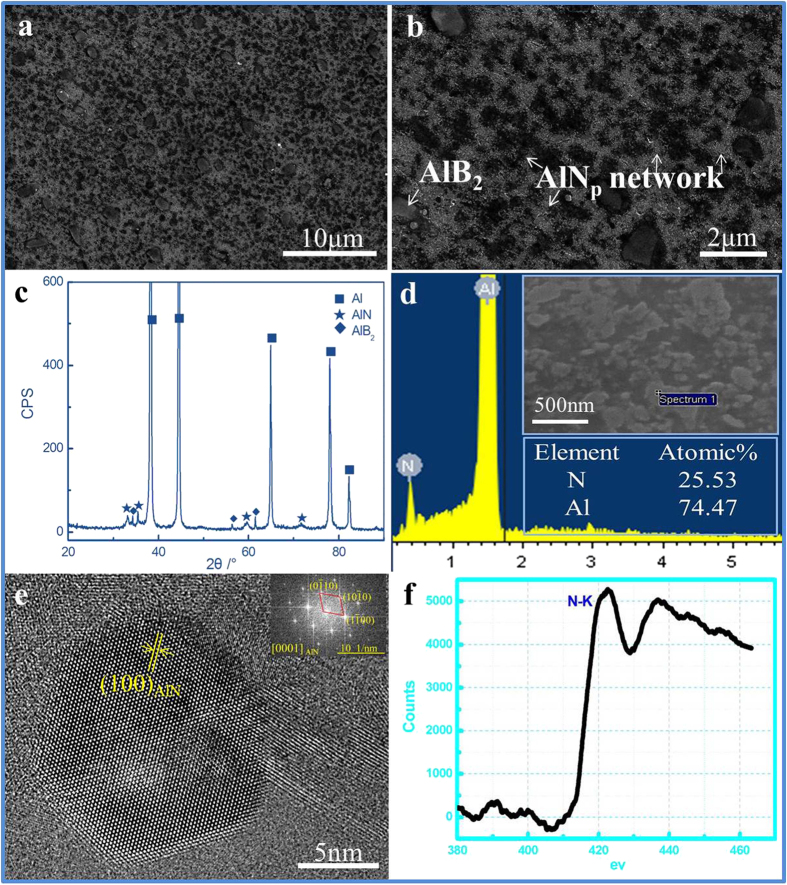
Phases identification and microstructures of the 16.4% AlN_p_/Al composites. (**a**,**b**) SEM images at low magnification show the typical dispersion of the phases. (**c**) XRD analysis reveals the main secondary phases: AlN and AlB_2_. The weaker peak amplitude of AlN is due to the low content. (**d**) EDS result of the irregularly gray particle is taken on the pointed dot in its top inset of 16.4% sample image. (**e**) HRTEM image of nano-scale AlN_p_ shows the clean interface with Al matrix. (**f**) EELS pattern that verifies the existence of N element in the particle shown in **e**.

According to the X-ray diffraction (XRD) curve in Fig. 1c, AlN (Hexagonal, P63mc) and AlB_2_ (Hexagonal, P6/mmm) were detected, which were further verified by energy dispersive spectroscopy (EDS) analysis (Fig. 1d). The weaker peak amplitude of AlN is because the content of AlN_p_ in the 16.4% AlN_p_/Al sample is low. Irregularly gray AlN_p_ with the size of 10~100 nm and the nearly hexagonal blocky AlB_2_ with size of 1~3 μm distributed homogenously in Al matrix, as shown in Fig. 1a,b.

Electron diffraction result (Fig. 1e) of AlN_p_ exhibits a typical diffraction pattern of [0001] zone axis, while electron energy loss spectroscopy (EELS) (Fig. 1f) proves the existence of N element in this particle. High-Resolution transmission electron microscope (HRTEM) image further proves that the *in-situ* synthesized AlN_p_ embedded in the matrix with a clean and close AlN_p_/Al interface with atom bonding. This can be attributed to the method we adopted, which avoid the oxidation and hydrolysis of AlN_p_. The well-defined interface between AlN_p_ and Al matrix can effectively transfer the mechanical load from matrix to ceramic particles. Due to the super thermal stability of AlN_p_, the interfaces with Al matrix have no pernicious reaction even at high temperatures. Moreover, the nanometric AlN_p_ tends to provide superior properties than the bulk ceramic^32^.

### Properties of the *in-situ* synthesized AlN_p_ reinforced Al matrix composites

To determine the strengthening effect of AlN_p_, the mechanical properties of the composites from RT to elevated temperatures have been tested.

**Figure 2:**
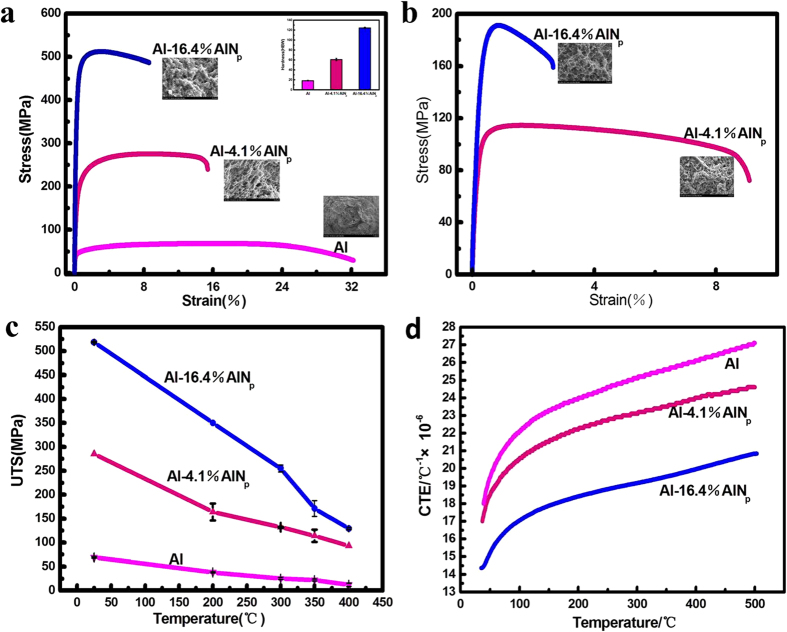
Properties of the AlN_p_/Al composites from RT to high temperatures. (**a**) Stress-strain curves, Brinell hardness (inset) and fracture surface images (inset) at RT. As for Brinell hardness results, each value was an average of at least four separate measurements taken at random places on the surface of specimens. (**b**) Stress-strain curves and fracture surface images (inset) of 4.1%, 16.4% AlN_p_/Al composites at 350 °C. (**c**) The UTS of AlN_p_/Al composites from room temperature to 400 °C. In each case, the average data was acquired from at least four specimens. (**d**) The linear thermal expansion behaviors of the samples with different contents of AlN_p_.

The mechanical properties of three samples at RT are presented in Fig. 2a, which displays that the UTS and hardness of the AlN_p_/Al composites increased rapidly with higher AlN_p_ contents. The tensile strength and hardness of 16.4 wt.% AlN_p_/Al (all compositions quoted in this work are in wt.% unless otherwise stated) are up to 518MPa and 124 HBW, respectively, 6 times higher than those of pure Al. Besides, the 16.4% AlN_p_/Al composite yields at 460MPa, while the sample without nanoparticles does only at 42MPa. Moreover, the elongation of 16.4% AlN_p_/Al can be kept at 9.5%.

In order to meet the requirement for heat resistance materials, the properties of the composites at high temperatures were also investigated, as shown in Fig. 2b–d. It is found that the UTS of samples are markedly elevated by *in-situ* synthesized AlN_p_. At 350 °C, the UTS of composites are all above 110MPa. With increasing AlN_p_ amount, UTS of 16.4% AlN_p_/Al can even achieve as high as 190MPa (Fig. 2b). The influence of AlN_p_ on thermal expansion behavior of the composites has been demonstrated in Fig. 2d. Due to the high strength and low linear expansion factor of AlN_p_ at temperatures ranging from RT to 500 °C, the expansion coefficient of the aluminum matrix is limited, thereby the expansion behavior of the composites has been restricted by the higher dimensional stability. The linear expansion coefficient of the 16.4% AlN_p_/Al composite is 19.5 × 10^−6^ K^−1^ at 350 °C. Under the same testing condition, the value of Al is 25.6 × 10^−6^ K^−1^, which is 31% higher than that of 16.4% AlN_p_/Al composite.

As shown above, the fabricated Al-16.4% AlN_p_ composite possesses excellent properties especially at high temperatures. The value is as high as 171MPa at 350 °C, much higher than the common heat resistant Al-Si-Cu alloy^9^. This kind of material has paved a possible way to improve the high temperature mechanical properties.

Based on the experimental data we got, there are mainly two reasons for the fantastic performance of the AlN_p_/Al composite at high temperatures: one is the high thermal stability of AlN_p_ (at high temperature it can also perform as nano scale hard ceramic); the other one is related to the spatial distribution of AlN_p_ throughout the Al matrix.

To study the formation of nano scale AlN_p_, the reaction mechanism in 16.4% AlN_p_/Al composite system has been investigated. At early stage, the initial interfacial reaction is described as follows^32^:


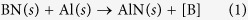


**Figure 3:**
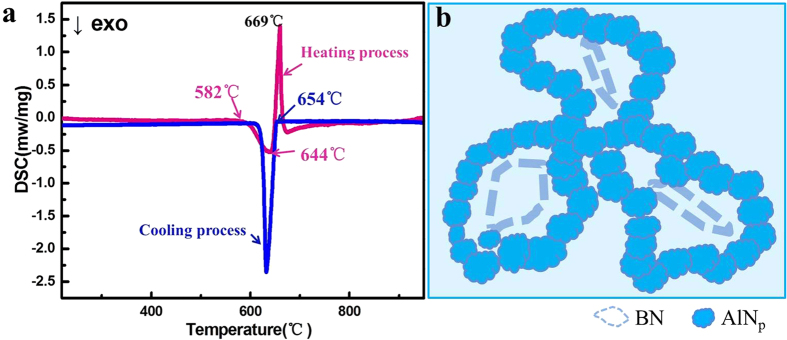
The formation of network structure in AlN_p_/Al composite. (**a**) DSC analysis of the 16.4% sample. (**b**) A network formation schematic: forming a closed and interconnected AlN_p_ circle in spatial.

Differential scanning calorimetry (DSC) analysis of the AlN_p_/Al composite system was conducted and the results show that there is an exothermal reaction between Al and BN starting at 580 °C in the heating curve as shown in Fig. 3, which is correspond to the formation of AlN_p_. That is to say, AlN_p_ is generated through solid-solid reaction. Considering the low solid solubility and the slow diffusion rate of N atoms in solid Al, AlN_p_ tends to be small and forming near the raw material BN in this circumstance. Then, at around 660 °C, there is an endothermic peak for Al melting with a more evident peak. As temperature continues to increase, the nano scale particles become bigger and cling to each other forming a closed and distorted circle in spatial (Fig. 3b). These spatial circles form a network structure throughout Al matrix.

At 800 °C, the boron atoms dissolve into aluminum to make up about 2.2% of the aluminum, as shown in Al-B phase diagram. When below 1030 °C, the remaining boron atoms form AlB_2_ following the expression^32^:


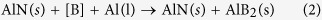


When cooled down, there is only the exothermal peak of Al solidification, proving that the reaction products are thermally stable during this process. The DSC is in accordance with the reaction mechanism described above.

**Figure 4:**
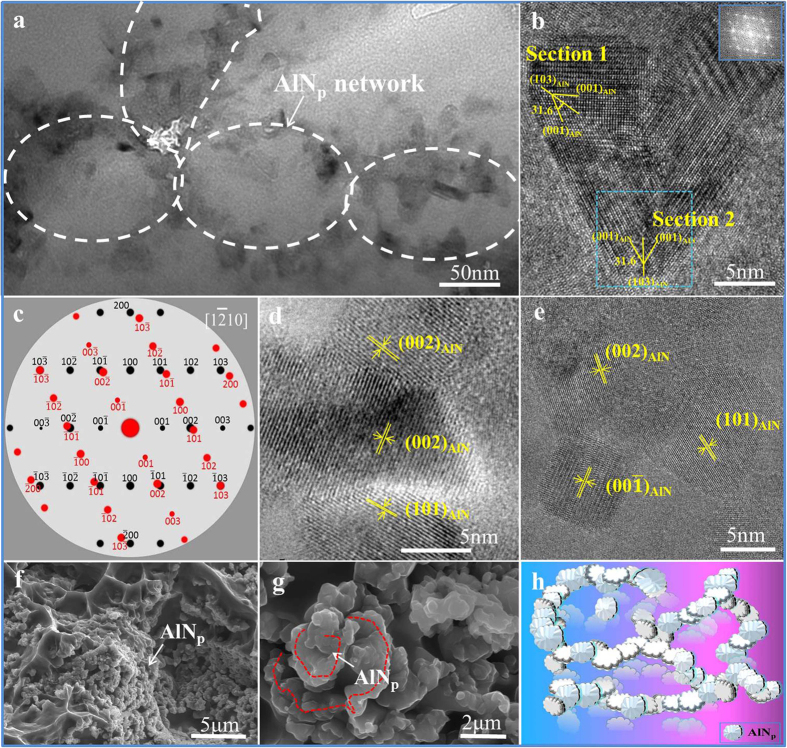
The 3D AlN_p_ network in Al matrix. (**a**) HRTEM image of the network structure: forming closed and interconnected circles in Al matrix. (**b**–**e**) HRTEM images of the typical conjunction mode between AlN_p_ in the network. The twinning conjunction mode: HRTEM images (**b**) and electron diffraction spots (**c**) for 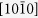 AlN zone axis of 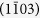 twin. Other conjunction mode: (**d,e**) the atomic bonding between AlN_p_ in the network structure as shown in **a**. (**f,g**) 3D morphology of AlN_p_ network on the fracture surface of the composite synthesized by large amount experiment. The experiment was conducted on the 16.4% AlN_p_/Al composites with pretreatment and the testing temperature was at room temperature. (**h**) A schematic of the 3D network of AlN_p_ proposed based on the experimental results of SEM, fracture surface and HRTEM analysis. The color changes from blue to red means the structure remains stable from RT to high temperature.

*In-situ* synthesized AlN_p_ and AlB_2_ fabricated in the matrix are thermodynamically stable, avoiding the wettability or aggregation problem as well as the reaction with H_2_O and O_2_. Thus, the interfaces of AlN_p_ are clean and connected through atomic bonding (Fig. 4b–e).

The typical morphology of the nano scale AlN_p_ with orientation of [0001] zone axis is hexagonal flake (Fig. 1e). The reason why the morphology shown in SEM images (Fig. 1a,b) is irregular is deduced that there is a certain conjunction among the nano scale AlN_p_, as proved in Fig. 4a. In order to find out the conjunction mode between AlN_p_, further analysis has been done. Figure 4b shows an exemplary conjunction in the AlN_p_/Al composite, which reveals that the two regions (section 1 and 2), separated by the boundary, characterizes a twin relationship. The electron diffraction shown in Fig. 4c is acquired from a selected area of section 2 marked by dotted line. The dominating diffraction spots with the incident beam parallel to the 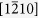 direction can be separated into two groups, which are rotated 63.26° to each other along the 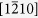 direction (Fig. 4b). The two regions, separated by the twin boundary, are related to each other by 180° rotation along the 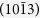 plane. Besides, for the P6_3_mc structure of AlN, the {000*l*} and 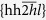 reflections with *l* = odd are forbidden^33^. The appearance of the {000*l*} forbidden reflection with *l* = odd in the 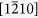 pattern can be attributed to double diffraction. For example, the combination of 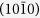 and 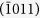 can give rise to a (0001) reflection when the incident beam is parallel to the 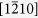 zone axis. Thus the twin boundary is a 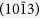 plane. Due to the twinning interlink, large amount of AlN_p_ are connected to each other by atomic bonding. This circumstance is common in the conjunction in AlN_p_/Al composite fabricated in this work. Thus far, the conjunction of AlN_p_ in nano chains is in atomic bonding and most of them are twinning.

As demonstrated in previous works^7^,^9^,^11^, the mechanical properties for particle reinforced composites will shift from brittle to ductile when temperatures are above 300 °C, leading to an increase of elongation. There is an anomalous phenomenon in this work: the elongation decreased with elevated temperatures. As can be seen from Fig. 2, the elongation of 16.4% AlN_p_/Al is 8% at RT and 3% at 350 °C. Based on the above solid-solid reaction mechanism and the outstanding mechanical properties at elevated temperatures, the spatial distribution of AlN_p_ has also been investigated. Through the observation of the fracture surface of the 16.4% AlN_p_/Al composite, the nano chains of AlN_p_ seem to be connected with each other forming a spatial structure throughout the Al matrix. However, SEM images are hard to reveal this clearly because of the nano scale of AlN_p_ chains. In order to get a better understanding of the spatial structure of AlN_p_ in the Al matrix, large amount of experiments were done. The AlN_p_ on the fracture surface in Fig. 4f,g are consistent with the irregular morphology in Fig. 1. Thus a schematic representation of the 3D network of AlN_p_ has been proposed by integrating SEM, fracture surface and HRTEM analysis (Fig. 4h). The color changes from blue to red means the structure remains stable from RT to high temperature. In general, they are connected to each other forming chains and build up a network of AlN_p_ in 3D direction, which support the Al matrix like the skeleton to human body.

## Discussion

In summary, the 3D network throughout the composites makes the soft Al matrix surrounded and strengthened by the *in-situ* synthesized hard AlN_p_ framework, which is consistent with the H-S upper bounds. The network structure acts as the hard armour for the soft Al matrix in the AlN_p_/Al composites, hindering the propagation of cracks. Also, there is a synergistic strengthening effect - reinforcement by the *in-situ* nano AlN_p_ and reinforcement by the 3D network structure of AlN_p_. The well interfacial bonding in AlN_p_/Al composite helps to transfer the stress homogeneously and avoid stress concentration. Such kind of framework shows good resistance to slip, as the stress required to push the dislocations through the particles barriers is high. The composite begins to yield when the stress is sufficient for the network barriers.

On the one hand, the nano chains of the network help to refine the aluminum grains while the soft aluminum around the hard AlN_p_ network can improve the ductility. All of these aspects lead to a high performance of the composite during a wide range of temperatures.

The 3D AlN_p_ network-reinforced Al matrix composites have a promising future. When the external stress is applied to the composite, the AlN_p_ network can effectively release the stress and powerfully impede the movement of the dislocations. In order to have a better understanding of the strengthening behavior of the 3D AlN_p_ network, the fracture characteristics of the AlN_p_/Al composite has been investigated. Due to the differences in load bearing temperature circumstance, the fracture characteristics showed distinct differences. The following discussion would focus on the typical temperatures of RT and 350 °C.

**Figure 5:**
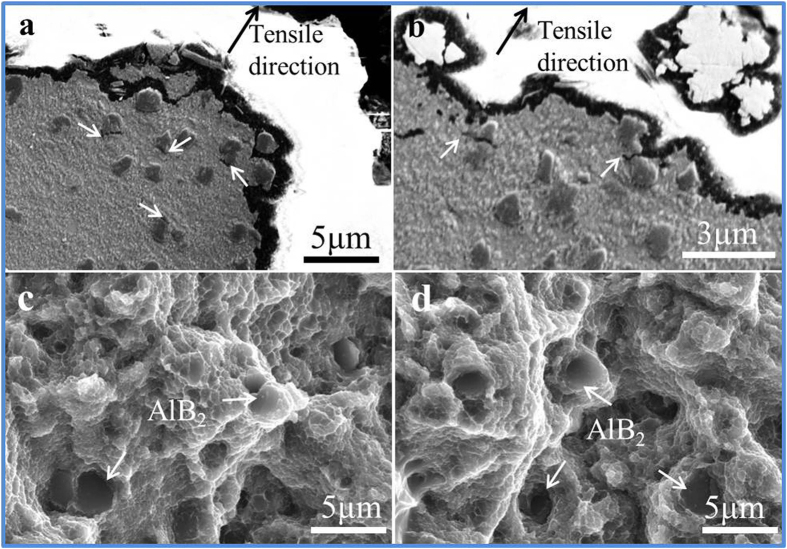
The fracture characteristic of the AlN_p_/Al composites at RT. (**a**,**b**) Cross-sectional observation of the 16.4% sample fracture reflecting the crack traces. (**c,d**) The fracture surface of the 4.1% (**c**) and 16.4% (**d**) composites shows the crack mode is particle crack. The exposed AlB_2_-terraces is the evidence.

Because of the combination of soft aluminum matrix and the *in-situ* fabricated hard 3D AlN_p_ network at RT, the composites can exhibit high strength while in the meantime acquiring certain ductility. The twin-bonded network can effectively pin dislocation motions. When the composite is under tensile state, the dislocations would aggregate at the interfaces between AlN_p_ network and Al matrix. The atomic bonding interfaces can effectively transfer the stress to AlN_p_. Therefore, the Orowan stress of AlN_p_ can bear such great stress without initiating crack. While, the premature cracks could occur in the AlB_2_ interlayer (Fig. 5a), which introduce defects to the composites and further lead stress concentration to the tip of the crack. Followed by crack accumulation and the subsequent linkage^34^ in the matrix (Fig. 5b), yet the network of AlN_p_ could effectively change the spread direction of the cracks and hinder the spread of the cracks to some degree. As mentioned above, AlB_2_ particles are homogenously distributed throughout the matrix without aggregation, thus it would not provide an adverse path for cracking. Finally the premature crack would lead to fracture of the materials. As a result, the fracture microstructure shows numerous AlB_2_-terraces without appearance of AlN_p_. The higher content of the reinforcements, the more fractured AlB_2_-terraces would appear (Fig. 5c,d). The AlB_2_-terraces also indicate that the interfacial atomic bonding between Al matrix and the secondary phases is strong enough to overcome the stress concentration. Because of the fracture mode, the spread rate of the crack is slow, which is good for higher reliability of the material.

**Figure 6:**
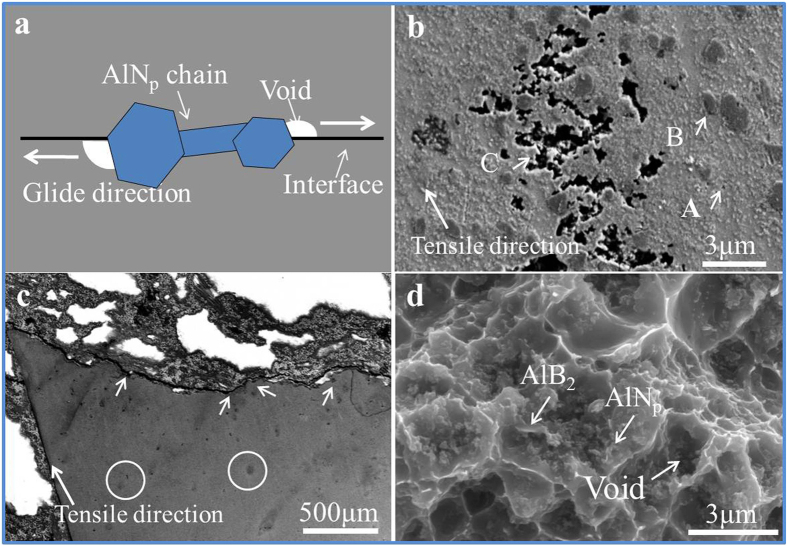
The fracture characteristic of 16.4% AlN_p_/Al composites at 350 °C. (**a**) The sketch map of the voids initiating at interface between AlN_p_ and the Al matrix mainly because of the discordant deformation speed at high temperature. (**b**,**c**) Cross-sectional observation of fracture helps to find out the trace of crack. (**d**) The fracture surface shows AlN_p_ network is the main load-bearing structure.

When the tensile test is performed at 350 °C, Al matrix becomes softer and has little resistance for the dislocation slip. While, the hard AlN_p_ can effectively hinder the movement of dislocations and the hard 3D network can strengthen Al matrix like the skeleton to human body. When the external stress is imposed on the AlN_p_/Al composite, Al matrix and AlN_p_ network performs differently. The soft Al matrix will have plastic deformation to offset the external stress. The network structure remains stable for hard strength and thermal stability of AlN_p_ and can effectively hinder the dislocation movement. With the increased stress, the soft aluminum will produce much more plastic deformation. While at the meantime, the network structure still remains no change. The discordant speed of deformation lead to the void initiated at the interface between AlN_p_ and Al matrix^35^ along the tensile direction as shown in Fig. 6a. Point A and B in Fig. 6b are the experiment results showing the initiated voids corresponded to the discussion before. It wouldn’t cause crack immediately for the high strength network of AlN_p_ and the ductility of aluminum matrix. Then with the increased time and stress, the 3D network of AlN_p_ suffers nearly all of the external stress along with the propagation and aggregation of the voids along the chain like point C shows in Fig. 6b. The amount of the void-zone continues to increase, and finally it will lead to fracture of the material. Figure 6c is the cross-sectional observation of the 16.4% AlN_p_/Al fracture at 350 °C, the void is more concentrated and close to the fracture. The voids are generated along the interface of AlN_p_, so there are plenty of AlN_p_ exposed. Besides, there are some deep black holes in the fracture (Fig. 6d), which correspond to the void zone as shown in Fig. 6a–c.

In conclusion, a 3D network of nano scale AlN_p_ has been successfully *in-situ* built by a liquid-solid reaction method in the composites, leading to the observed increase in strength, especially at high temperatures. The ultimate tensile strengths of 16.4% AlN_p_/Al can be up to 518MPa at RT and 190MPa at 350 °C. The novel composites fabricated in this work may contribute to designing high-performance heat resistance materials for advanced structural applications.

## Methods

### AlN_p_/Al composites fabrication

The raw materials used in this work contain commercial Al powders (99.7%), hexagonal Boron Nitride powders (98.5%) and active carbon powders (99.0%). The mixture of powders was consolidated under Argon gas by liquid-solid reaction, and then the obtained ingot was extruded at about 500 °C with an extrusion ratio of 20:1, according to the CN105385902A patent. For convenience, the AlN_p_ reinforced Al matrix composites are defined as 4.1% AlN_p_/Al, 16.4% AlN_p_/Al with different fractions of reinforcement particles in this work. The raw powders used for the large amount experiment was pretreamented.

### Phase identification and microstructural characterization

X-ray diffraction (XRD, Rigaku D/max-rB) was used to identify the phases contained in the AlN_p_/Al composites. Phases identification and microstructures characteristic of the AlN_p_/Al composites were performed utilizing field emission scanning electron microscope (FESEM, model SU-70, Japan) equipped with an energy dispersive spectroscopy (EDS) detector and High-Resolution transmission electron microscope (HRTEM, ZEISS LIBRA200) assembled with electron energy loss spectroscopy (EELS). Thermal stability of the composites was investigated by means NETZSCH DSC 404C and NETZSCH DIL 402 C high temperature dilatometer at a heating rate of 10 K/min.

### Mechanical property testing

The hardness was measured on a HB-3000C Brinell hardenss tester with parameters of HB5/250/15. Each value was an average of at least four separate measurements taken at random places on the surface of specimens. Tensile testing was conducted on the extruded composites after T2 heat treatment (250 °C, 3h) at temperatures of RT, 200 °C, 300 °C, 350 °C and 400 °C. These tests were conducted by assuring the specimens to stabilize at temperatures for about 30minutes prior to test using an extension rate of 2 mm/min, and the matrix alloy were also measured for comparison. In each case, the average data was acquired from at least four specimens. The large amount experiment was conducted on the AlN_p_/Al composite with pretreatment. The testing temperature was at room temperature.

## Additional Information

**How to cite this article**: Ma, X. *et al.* A novel Al matrix composite reinforced by nano-AlN_p_ network. *Sci. Rep.* **6**, 34919; doi: 10.1038/srep34919 (2016).
